# Authors’ response: Autochthonous transmission patterns of dengue virus serotype 2 in Italy: evidence from outbreaks in 2024

**DOI:** 10.2807/1560-7917.ES.2026.31.34.2600721

**Published:** 2026-08-27

**Authors:** Carla Molina Grané, Martina Del Manso, Patrizio Pezzotti, Stefano Merler, Piero Poletti

**Affiliations:** 1 Center for Health Emergencies, Fondazione Bruno Kessler, Trento, Italy; 2 Department of Infectious Diseases, Istituto Superiore di Sanità, Rome, Italy

**Keywords:** dengue, autochthonous, outbreak, Europe, Italy, transmission, transmission chain reconstruction, model

**To the Editor:** We thank Aguiar and colleagues for their interest in our study on the transmission patterns of dengue virus (DENV) serotype 2 in Italy [1], and for expanding on the concept of a near-critical transition of dengue transmission in Europe. However, we wish to clarify some points regarding our methodology and the interpretation of our findings.

We emphasise that our study does not characterise DENV transmission as persistently or homogeneously supercritical in Italy. Rather, our use of the time-varying reproduction number (R_t_) was intended to capture the transient and heterogeneous nature of DENV transmission in non-endemic settings. The results clearly show that R_t_ briefly peaked above one and varied significantly across outbreaks and provinces [1]. In a previous study we have already shown that DENV transmissibility in Italy is strongly heterogeneous and generally low, exceeding the epidemic threshold only for a limited period, with most areas at risk of onward transmission for less than 2 generation times [2]. Therefore, we agree that it is certainly plausible that the emergence of small clusters of cases may occasionally result from the convergence of multiple factors, including stochastic effects. However, with more than 200 reported cases distributed over 3 months and a high level of transmissibility [1,3], the Fano outbreak cannot simply be explained by stochastic amplification of a low transmission risk. Rather, it reflects the convergence of seasonal, environmental and epidemiological conditions that were highly favourable to transmission.

Regarding the outbreak resolution, our analysis did not conclude that vector control measures alone were responsible for the decline in transmissibility. We explicitly sought to disentangle the relative contributions of various factors. We showed that control interventions were associated with a mean reduction of ca 40% in transmission, and 1°C rise in average temperature – a proxy for vector abundance – was associated with ca 20% mean increase in transmission [1]. This highlights that the decline in transmissibility was a multifactorial process. However, from a public health perspective, we believe that emphasising the importance of early detection and timely interventions (including raising awareness, active case finding and vector control measures) in reducing transmission is crucial. This analysis [1], combined with that of previous outbreaks [2-4], clearly shows – consistently across outbreaks – that both the number of cases and transmissibility begin to decline soon after an outbreak is detected. Our quantification of intervention effectiveness did not rely on macroscopic trends of the R_t_ trajectory, which may indeed be affected by delays in symptom onset data, but was derived from an individual-level regression model [1]. This model directly linked secondary transmission events from each potential infector to whether vector control had been implemented at their specific exposure site prior to symptom onset, while adjusting for age, sex, temperature, population density, vegetation and the time required for their reporting after symptom onset.

We agree that a single observed outbreak cannot describe the full probability distribution of introduction outcomes, including failed introductions. While our paper focused on gathering empirical evidence from the outbreaks observed in 2024 [1], in a previous study we have shown that between 2006 and 2023 only 93 cases were autochthonous while 1,435 DENV infections were reported as imported [2]. This unequivocally indicates that large outbreaks do not recur whenever the virus is introduced. In the same study, we also concluded that the precise location of historical outbreaks may have been determined by incidental case importation rather than by a localised higher risk of onward transmission. Therefore, observed outbreaks represent events that could probabilistically occur across many other suitable regions of the country.
